# Dissection of Targeting Molecular Mechanisms of Aristolochic Acid-induced Nephrotoxicity *via* a Combined Deconvolution Strategy of Chemoproteomics and Metabolomics

**DOI:** 10.7150/ijbs.69618

**Published:** 2022-02-21

**Authors:** Qian Zhang, Piao Luo, Jiayun Chen, Chuanbin Yang, Fei Xia, Junzhe Zhang, Huan Tang, Dandan Liu, Liwei Gu, Qiaoli Shi, Xueling He, Tong Yang, Jigang Wang

**Affiliations:** 1 Artemisinin research center, and Institute of Chinese Materia Medica, China Academy of Chinese Medical Sciences, Beijing 100700, China.; 2 School of Chinese Materia Medica, and State Key Laboratory of Component-based Chinese Medicine, Tianjin University of Traditional Chinese Medicine, Tianjin 301617, China.; 3 School of Traditional Chinese Medicine, Southern Medical University, Guangzhou 510515, China.; 4 Department of Urology, The Second Clinical Medical College, Jinan University (Shenzhen People's Hospital), Shenzhen, Guangdong 518020, China.; 5 Center for Reproductive Medicine, Dongguan Maternal and Child Health Care Hospital, Southern Medical University, Dongguan 523125, China.; 6 Central People's Hospital of Zhanjiang, Zhanjiang, Guangdong 524037, China.; 7 Guangdong Provincial Key Laboratory of New Drug Screening, School of Pharmaceutical Sciences, Southern Medical University, Guangzhou 510515, China.

**Keywords:** Aristolochic acid nephropathy, chemical proteomics, mitochondrial dysfunction, apoptosis, metabolism

## Abstract

Aristolochic acid (AA), mainly derived from herbal *Aristolochia* and* Asarum* plants, was listed as a human carcinogen class I in 2002. Aristolochic acid nephropathy (AAN) is a rapidly progressive tubulointerstitial nephritis and urothelial cancer caused by AA. However, the targeting molecular mechanisms of AAs-induced nephrotoxicity are largely unclear. This study aims to dissect targeting molecular mechanisms of AA-induced nephrotoxicity. Activity-based protein profiling (ABPP) in combination with cellular thermal shift assay (CETSA) was performed to identify the AAs binding target proteins. Our data indicated that several key enzymes in the metabolic process and mitochondrial respiration including IDH2 and MDH2 (Krebs cycle), PKM and LDH (aerobic respiration), FASN (fatty acid beta-oxidation), HK2 (glucose metabolism), and ATP synthase were identified as directly binding targets of AAs. Metabolomics and oxygen consumption rate (OCR) experiments further confirmed that AAs targeting proteins disrupted metabolic biosynthesis processes and impaired mitochondrial functions. Ultimately, AAs induced renal cells apoptosis by disturbing various biological processes. Cumulatively, AAs may directly bind to key proteins involved in the metabolic process and mitochondrial homeostasis, and finally induce aristolochic acid nephropathy. Our findings provide novel insight into underlying mechanisms of AAs-induced kidney toxicity, which may help to develop therapeutic strategies for AAN.

## Introduction

Aristolochic acid nephropathy (AAN), previously known as Chinese herb nephropathy (CHN), is a rapidly progressive tubulointerstitial nephritis causing end-stage renal disease and urothelial carcinoma 1. This nephropathy is mainly caused by aristolochic acids (mainly aristolochic acid I and aristolochic acid II) (AAs) 2, 3. AAs are a class of natural products mainly derived from herbal medicinal plants, such as *Aristolochia* and *Asarum* 4, 5. Epidemiological investigations indicated that people in the Balkans accidentally ingested AA-contaminated wheat and groundwater or mistakenly used it as a weight-loss drug, leading to Balkan endemic nephropathy (BEN) in 1990s 6-8. Later this similar etiology of renal disease and the upper tract urothelial cancer (UTUC) induced by AAs have emerged worldwide (mainly in East Asia and Southeast Asia) 9-11, which related to the fact that people in these areas are accustomed to consuming traditional Chinese medicine (TCM). Thus, the toxic effects induced by AAs have become a serious global public health problem. Due to the toxicity, AA was listed as a human carcinogen class I by the World Health Organization International Agency for Research on Cancer in 2002.

AAs preferentially target proximal tubular epithelial cells (PTEC) to induce nephrotoxicity 12. Nephrotoxicity caused by AAs has multiple mechanisms, including immune-inflammation, apoptosis, oxidative stress, hemodynamic abnormalities and endoplasmic reticulum stress 13-17. For instance, it was reported that AAs metabolites, aristolactam-nitrenium ions, react with DNA bases to form the covalent AA-DNA adducts 18, 19, thereby leading to nephrotoxic, genotoxic, and carcinogenic effects. However, the direct binding targets and exact molecular mechanisms of AA-induced nephrotoxicity are remaining unclear.

Chemical proteomics is a novel and promising approach that can systematically identify the covalent binding targets of compounds 20, 21. Previously, we utilized this approach to identify the underlying targeting mechanisms of many natural products, such as artemisinin, celastrol, curcumin and andrographolide 20-23. This contributes to further understanding the potential mechanisms of pharmacology and toxicology of natural products.

Here, a chemical proteomics method based on the activity-based protein profiling (ABPP) was used to comprehensively profile and identify the AAs' covalent binding targets *in vivo* and *in vitro*. A wealth of protein targets is involved in multiple important biological processes, especially mitochondrial metabolism and cellular respiration. Among them, several key enzymes were validated by cellular thermal shift assay (CETSA) and pull-down western blot experiments. Interestingly, the activities of target enzymes were impaired by AAs. Therefore, these results suggest that AAs may induce nephrotoxicity through a complex protein-targeting mechanism. In addition, we further validated the AAs targets involved in metabolic processes and mitochondrial dysfunction by using metabolomics, oxygen consumption rate (OCR) and mitochondrial membrane potential (MMP) assays. Our data support a promising strategy to better understand the molecular mechanisms of toxic compounds.

## Results

### Synthesis and bioactivity of aristolochic acid probe

**Scheme 1:**
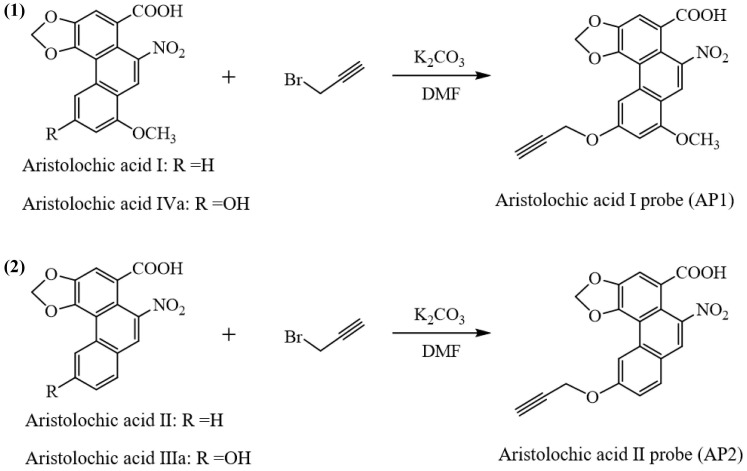
Procedures for the preparation of aristolochic acid probes. (**1**) Chemical structures of aristolochic acid I (AAI), Aristolochic acid IVa (AAIVa) and the alkyne-tagged-clickable probe (aristolochic acid I probe, AP1). (**2**) Chemical structures of aristolochic acid II (AAII), Aristolochic acid IIIa (AAIIIa) and the alkyne-tagged-clickable probe (aristolochic acid II probe, AP2).

**Figure 1:**
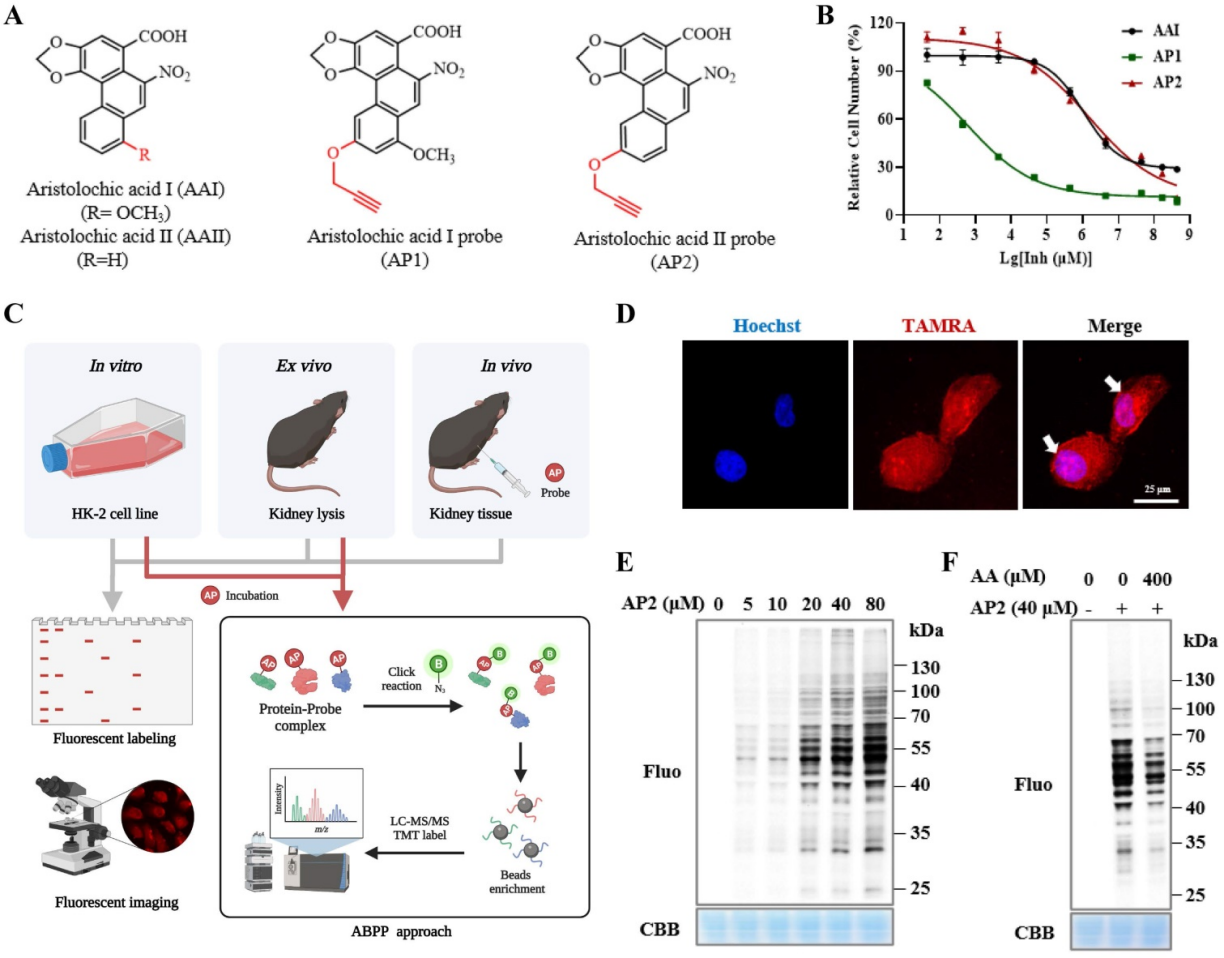
** Bioactivity and fluorescence labeling of aristolochic acid probe.** (**A**) Chemical structures of aristolochic acid I (AAI), aristolochic acid II (AAII), aristolochic acid probe 1 (AP1) and aristolochic acid probe 2 (AP2). (**B**) The toxicity effects of aristolochic acid probes (AP1, AP2) compared with aristolochic acid I (AAII) on HK-2 cell. (**C**) Schematic model showing the workflow of ABPP approach combining with bio-orthogonal click chemistry. (**D**) Fluorescent cellular imaging to track the subcellular location of the AP2 (40 µM) in HK-2 cells (scale bar = 25 µm). (**E**) *In situ* labeling protein in an AP2 dose-dependent manner in HK-2 cells. (**F**) The competition of labeling protein with AP2 by excess AA in HK-2 cells.

To identify AAs direct binding proteins using ABPP, two aristolochic acid probes (AP1, AP2) conjugated with a clickable alkyne tag respectively were chemically designed and synthesized (Figure 1A). The design and synthesis schemes of two AA-probes are shown in Scheme 1. We first compared the cell toxicity effect of AA-probes and AAs by using human renal proximal tubule epithelial cells (HK-2 cells). Our results showed that the toxicity effect of AP2 was as potent as the original compound, aristolochic acid I (AAⅠ) sodium salt, while AP1 displayed strong toxic effects (Figure 1B). However, the toxicity effect of two analogues (AAⅣa, AAⅢa) was significantly lower than AAⅠ (Figure S1A). Collectively, the probe AP2 has a similar toxicity profile to AAⅠ sodium salt. Thus, AP2 was selected for identified AAs' binding targets.

### Fluorescence labeling of aristolochic acid probe

To explore the cellular localization of AA-probe in HK-2 cells, live HK-2 cells were treated with AP2 probe for 4 h, washed with PBS, fixed with 4% paraformaldehyde and permeabilized with 0.2% Triton-100. Cells were then clicked with fluorescence dye and processed to image. Fluorescent signaling was scattered throughout the cell, indicating that AA-probe distributed in the nucleus and cytoplasm (Figure 1D), suggesting that AA-probe can bind to cell contents (nucleic acid or protein and so on). To a certain extent, this result is similar to previous findings that is the covalent formation of adducts with AAs 18. Next, to identify and profile the target proteins of AAs, an ABPP assay was carried out *in vitro*, *ex vivo* and *in vivo*. A common workflow of this ABPP approach 20, 21 is illustrated in Figure 1C.

Firstly, we optimally chose AA-probe (AP2) for further in-depth experiments by combining the toxicity effects of two probes on HK-2 cells and the results of pre-labeling target proteins experiment* in situ*. HK-2 cells were incubated with AA-probe and then crude proteins were extracted. Protein samples were processed by click chemistry and separated by SDS-PAGE gel electrophoresis. AA-probe binding to proteins could be visualized by a fluorescence imaging scanner. Many proteins were labeled by AP2 in a dose-dependent manner (Figure 1E). Furthermore, AP2 could bind to different targets that were competed away by pretreatment with AAI (Figure 1F). We also implemented cellular imaging fluorescence experiments *in situ*. Cellular fluorescence intensity varied in an AP2 dose-dependent manner (Figure S1B). Co-incubation with excess AAI could effectively compete with the labeling proteins of AP2 *in situ* (Figure S1C). Secondly, we conducted experiments similar to the above cellular experiment *ex vivo*. Proteins were extracted from the kidney of mice and then treated with AP2 via a click reaction. Kidney proteins were labeled by AP2 in a dose-dependent manner and could be competed away by an excess amount of AAI (Figure S1D-E). Moreover, similar cell labelling experiments were also carried out *in vivo*. Following tail vein injection of AP2, mouse kidneys were collected and extracted proteins were then subjected to click fluorescent labeling. Results showed that AP2 could also bind to many proteins *in vivo* (Figure S1F). In summary, these results indicate that the AP2 covalent binding targets are similar to aristolochic acid analogues.

### Identification of the nephrotoxic targets of aristolochic acid

**Figure 2:**
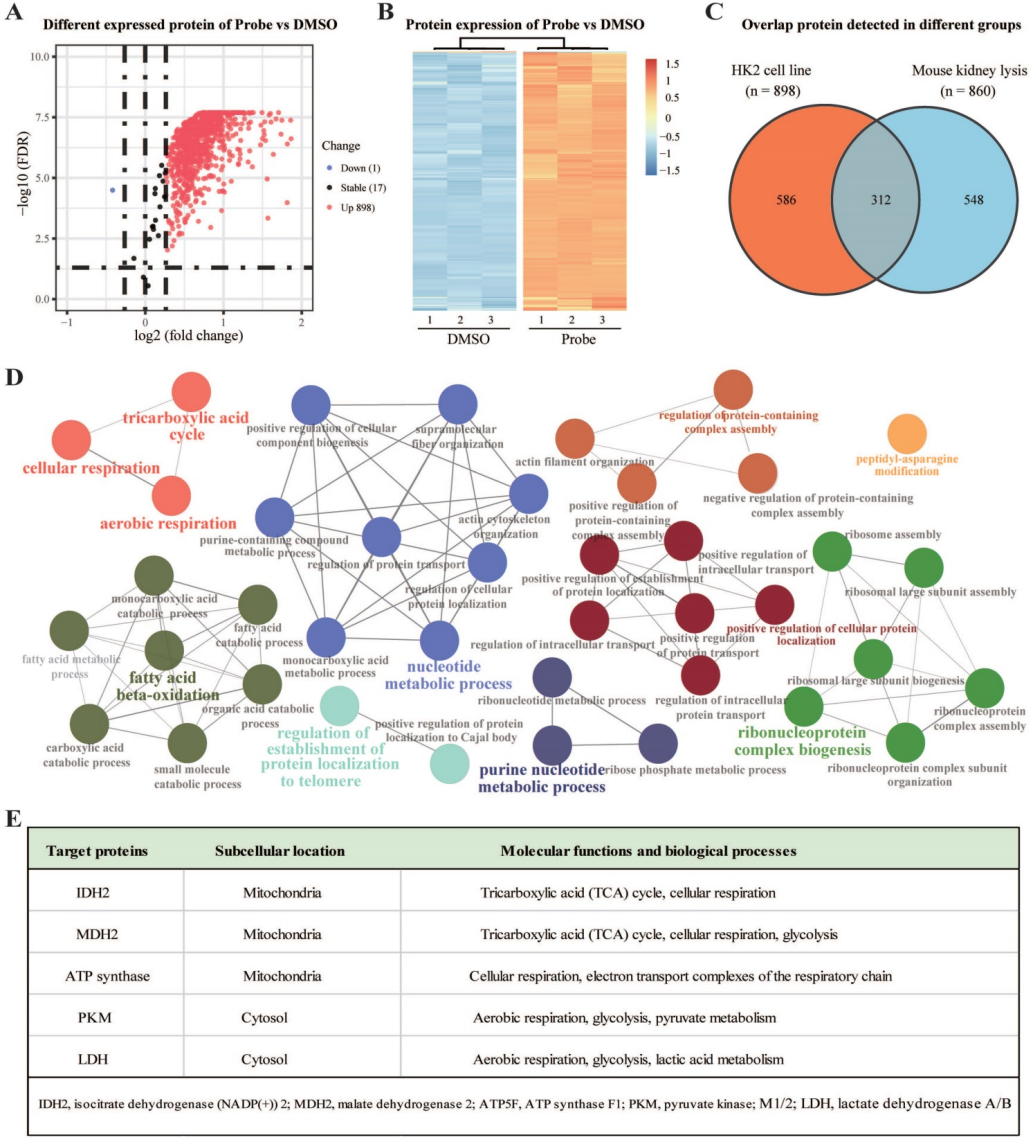
** Aristolochic acid targeting proteins participate in key metabolic processes in kidney.** (**A**) Volcano plot depicting the differential enrichment of proteins in AP2 (probe) vs DMSO groups. (**B**) Temporal patterns for AA targeting proteins (AP2 vs DMSO). (**C**) Venn digraph depicting the same target proteins of AA in kidney tissues and HK-2 cells. (**D**) Target proteins involved in essential biological processes by Gene Ontology (GO) analysis. (**E**) The representative AA targets and their biological functions.

Next, to identify the potential binding proteins of AA by ABPP approach, HK-2 cells and mouse kidney lysates were first incubated with AP2 for 4 h. Subsequently, proteins lysates were incubated with biotin-alkyne, and the AP2 binding targets were affinity-purified with streptavidin beads. Samples were digested to peptides and identified by LC-MS/MS according to our previous protocols 20. Compared to the DMSO group, a total of 898 target proteins were identified as direct binding targets of AAs in HK-2 cells (Figure 2A-B). Meanwhile, a total of 860 human homologous proteins were identified as direct binding targets of AAs in the lysate of mouse kidneys (Figure S2). Among these proteins, 312 proteins were identified both in HK-2 cells and mouse kidney (Figure 2C). Gene ontology (GO) analysis for these 312 shared targets indicated that these proteins participate in key biological processes, including tricarboxylic acid (TCA) cycle, aerobic respiration, fatty acid beta-oxidation, nucleotide metabolic process and purine nucleotide metabolic process (Figure 2D). Interestingly, these metabolism-related target proteins were mainly located on the mitochondria or cytosol (Figure 2E). Thus, AAs may induce nephrotoxicity via direct targeting mitochondrial proteins to affect key metabolic processes such as TCA cycle (Krebs cycle), aerobic respiration, and cellular respiration.

### Validation of the nephrotoxic targets of aristolochic acid

**Figure 3:**
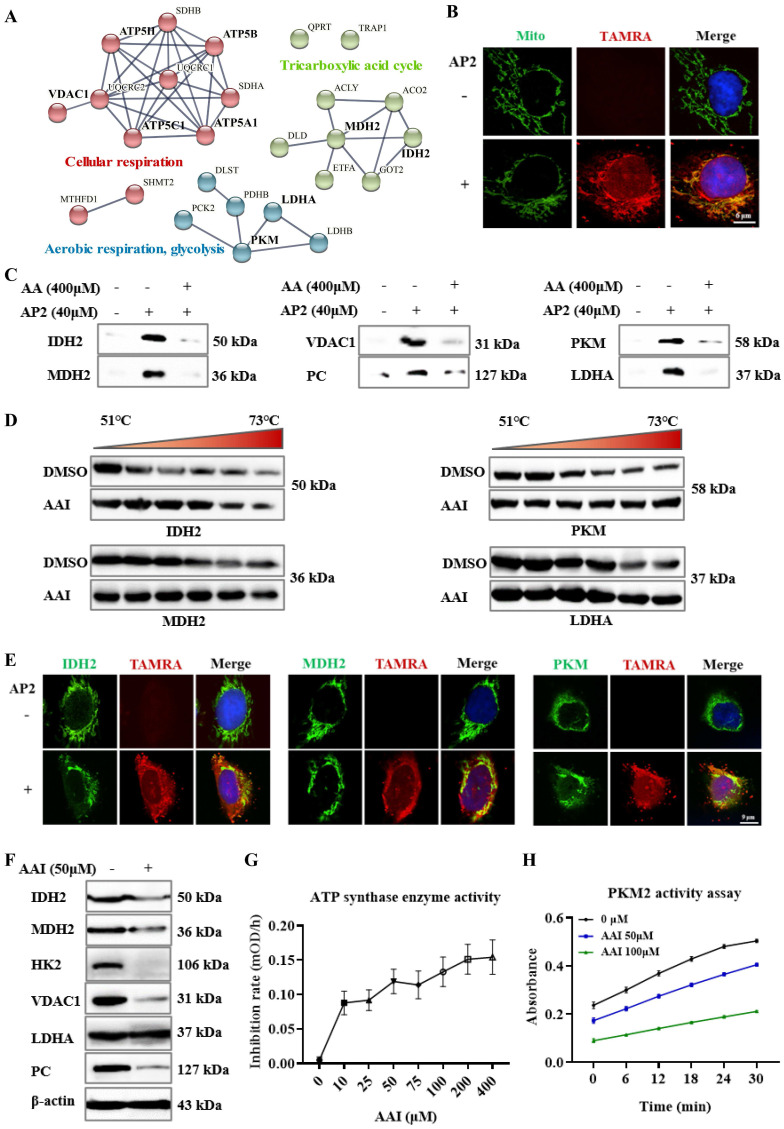
** Aristolochic acid targets multiple mitochondrial proteins and metabolic enzymes.** (**A**) The PPI analysis of AA targets involved in tricarboxylic acid (TCA) cycle and respiration processes. (**B**) Fluorescence co-localization of AA-probe (a red fluorescence dye) with mitochondria (green) in HK-2 cells by using confocal microscopy to image, scale bar = 6 µm. (**C**) Pull-down western blotting study to verify AA directly targeting IDH2, MDH2, VDAC1, PC, PKM and LDHA proteins. (**D**) CETSA-WB experiment to validate the AA binding to IDH2, MDH2, PKM and LDHA proteins. (**E**) Fluorescence staining of target proteins (green) and AA-probe (a red fluorescence dye), scale bar = 9 µm. (**F**) Expression of IDH2, MDH2, HK2, VDAC1, PC and LDHA proteins in AA treatment or DMSO group. (**G**) The ATP synthase enzyme activity with or without AAs in the lysate of kidney. (**H**) The PKM2 activity of rhPKM2 with or without AAs.

We next conducted protein and protein interaction (PPI) analysis for AA targets involved in key metabolic processes TCA cycle and respiration processes (Figure 3A), as indicated in the GO analysis. Of interest, several rate-limiting enzymes occupied an important position in PPI analysis, such as isocitrate dehydrogenase 2 (IDH2), malate dehydrogenase 2 (MDH2), ATP synthase, pyruvate kinase M1/2 (PKM), and lactate dehydrogenase A/B (LDH). Therefore, these targets were selected for verification of their bind with AP2 *in vitro*. Since these enzymes are mainly distributed in mitochondria. Here, we first used a red fluorescence dye of AA-probe (AP2) to track its cellular co-localization with mito-tracker green, a mitochondrion-selective. As expected, the fluorescence imaging confirmed the co-localization of AP2 with mitochondria in HK-2 cells (Figure 3B). Thus, AAs may target multiple mitochondria proteins.

A pull-down experiment was then used to validate the AP2 binding to these targets in the kidney lysates. The results indicated that AP2 effectively pulled down IDH2, MDH2, pyruvate carboxylase (PC), voltage-dependent anion-selective channel protein 1 (VDAC1), PKM and LDHA (Figure 3C). Meanwhile, the binding of AP2 to these targets could be competed against by AAs (Figure 3C), further suggesting that these proteins may be direct targets of AAs. We also validated that AAs targeted several selected metabolic enzymes and mitochondria-related proteins by pull-down experiment, which include acetyl-CoA carboxylase 1 (ACACA), ATP synthase, fatty acid synthase (FASN) and hexokinase-2 (HK2) (Figure S3A). The CETSA based on the thermodynamic stabilization of protein is regarded as an effective approach for monitoring the compound binding to protein 24. CETSA-WB experiment in kidney lysate was also performed to validate the direct binding proteins to AAs. Our results showed that IDH2, MDH2, PKM, LDHA, HK2, VDAC1 and PC all occupied more significant thermal stabilization in the AAI treatment group (Figure 3D, Figure S3B). Moreover, the immunofluorescence staining confirmed the co-localization of AP2 with IDH2, MDH2, ATP synthase or PKM in HK-2 cells (Figure 3E, Figure S3C). To verify the interaction of AAI-target proteins, we carried out molecular docking analysis on the solved structures of HK2, PKM, LDHA (glucose metabolism), ATP synthase, IDH2, MDH2 (TCA cycle), FASN (fatty acid metabolism) and VDAC1 proteins. The analysis results suggested the interactions between AAI and these representative target proteins (Figure S4A-H, supporting information).

In addition, western blot assays indicated that the expressions of IDH2, MDH2, HK2, VDAC1 and PC were down-regulated in AA-treated kidneys, but not LDHA compared with DMSO group (Figure 3F), further supporting the notion that AAs affect key metabolic processes. ATP synthase and PKM are crucial enzymes involved in glucose metabolism, ATP production and cellular respiration. Enzyme activity assays showed that AAs inhibit their enzyme catalytic activities (Figure 3G-H). Collectively, we systematically profiled and identified the targets of AAs in HK-2 cells and kidneys by a novel ABPP approach combining with CETSA, revealing that AAs may cause nephrotoxicity via targeting multiple key metabolic enzymes.

### Metabolomic analysis of aristolochic acid in kidney and serum

**Figure 4:**
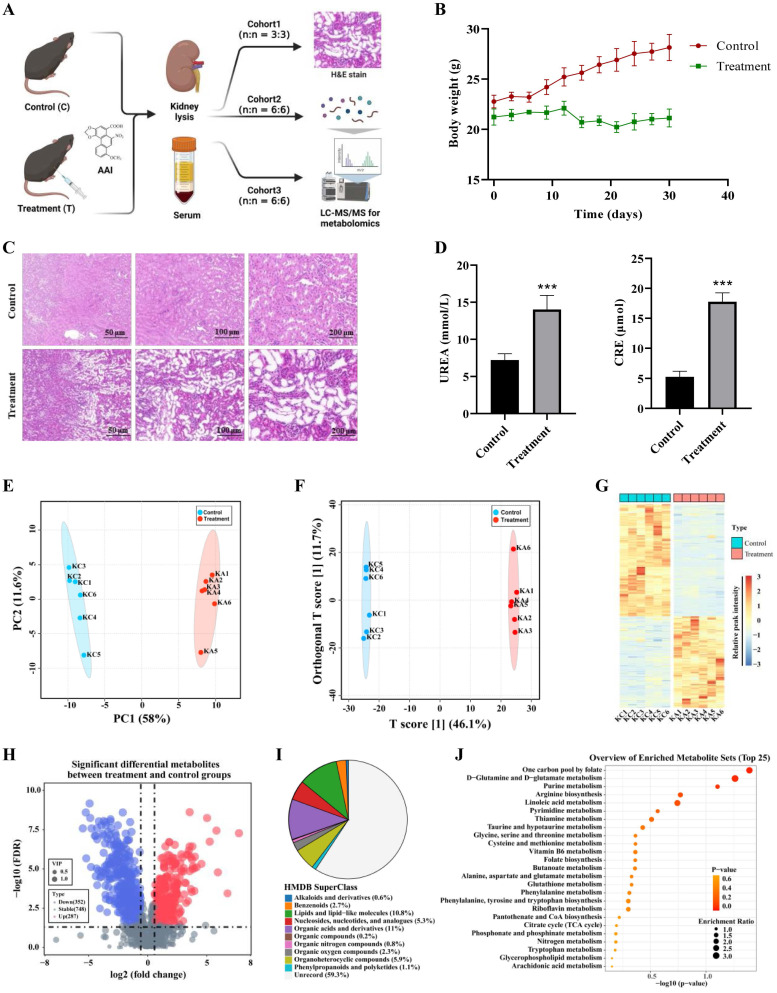
** Metabolomics reveals AA-induced nephrotoxicity mainly through purine metabolism, amino acids metabolism and TCA cycle.** (**A**) The strategy for metabolomics on AAI-induced nephrotoxicity. (**B**) Body weight from AAI-treated (treatment group) and control group mice. (**C**) Representative H&E staining in the kidneys of AAI-treated and control mice. (**D**) Biochemical indicators for UREA and CRE in the serum of mice. (**E**) The PCA plots of the DMSO and AAI-treatment groups in the kidney, *** *P* < 0.001, compared with control (n=5). (**F**) Ortho PLS-DA analysis of the DMSO and treatment groups in the kidney. (**G**) Heatmap showing top up and down metabolites in the kidney after AAI-treatment. (**H**) The volcano map displays the different metabolites in the kidney after AAI-treatment. Up-regulated metabolites were represented by red dots, down-regulated metabolites were represented by blue dots. (**I**) Classification information of HMDB database annotations in the kidney after AAI-treatment. (**J**) KEGG biochemical metabolic pathway and signal transduction pathway in the kidney after AAI-treatment.

To further interrogate the impairment of metabolic processes after exposure to AAs *in vivo*, AA-induced nephrotoxicity mouse model was established by intraperitoneal injection with AAⅠ. The basic parameters of the animal were monitored to evaluate whether AAI caused kidney injury in our study (Figure 4A). Compared to the control group, AAI not only significantly reduced body weight, but also resulted in an increase of the kidney/body weight ratio (Figure 4B, Figure S5B). AAI caused kidney injury and morphological changes (Figure 4C, Figure S5A, Figure S5B), as evidenced by aggravating hemorrhagic necrosis across the cortex and tubules in H&E staining images. AAI also disordered changes in biochemical parameters, such as UREA and creatinine (CRE) (Figure 4D). Routine blood tests showed that the treatment group had signs of body weakness (Figure S5C). These results indicated AAI-induced nephrotoxic effects in mice.

A nontargeted metabolomics method in kidney tissues and serum of DMSO and AAI-treated mice was further used to characterize AAI-induced toxicity. A total of 1387 metabolites were detected in both positive and negative ion modes by UHPLC-MS/MS technique. The pearson correlation coefficient was analyzed between quality control (QC) samples. The higher correlation of QC kidney tissues (the R^2^ value close to 1) showed that the stability and quality of the whole detection process was better in both positive or negative ion modes (Figure S6A-B). Next, all metabolites combined with both positive and negative ion modes for the kidney tissues were analyzed by principal component analysis (PCA), indicating that there were significant differences in the DMSO and AAI-treatment group (Figure 4E). Orthogonal partial least squares-discriminant analysis (Ortho PLS-DA) also showed that metabolites were the separation between the DMSO and treatment group (Figure 4F). Altogether, these results suggest that the classification model is stable and reliable.

Next, differential metabolites in kidneys after AAI-treatment were identified and shown in in a heatmap (Figure 4G). Volcano map depictions were used to visually display the overall distribution of different metabolites (Figure 4H). The abscissa represented the change in the fold change of metabolites in different groups (log_2_FoldChange), and the ordinate represented the significance level of difference (-log10 p-value). Each point in the volcano map represents a metabolite. The 287 significantly up-regulated metabolites were represented by red dots, the 352 significantly down-regulated metabolites were represented by blue dots, and the size of the dot represented the VIP value. In addition, we noted metabolites by human metabolome database (HMDB) classification. Results showed that changed metabolites after AAI-treatment were mainly organic acids and derivatives, lipids and lipid-like molecules, and organoheterocyclic compounds (Figure 4I). Subsequently, we performed KEGG enrichment annotation analysis on the differential metabolites, suggesting that AAI may affect purine metabolism, arginine biosynthesis, amino acids metabolism and TCA cycle in kidney (Figure 4J). Moreover, metabolomics studies in serum of mice after AAI-treatment were also analyzed and showed similar results to the kidney tissue as mentioned above (Figure S6C-I). Overall, metabolomic results in the kidney tissues and the serum indicated that the AAI impairs key metabolic pathways, which is consistent with our ABPP results as mentioned above.

### Metabolic dysfunction caused by aristolochic acid via inducing mitochondrial damage

**Figure 5:**
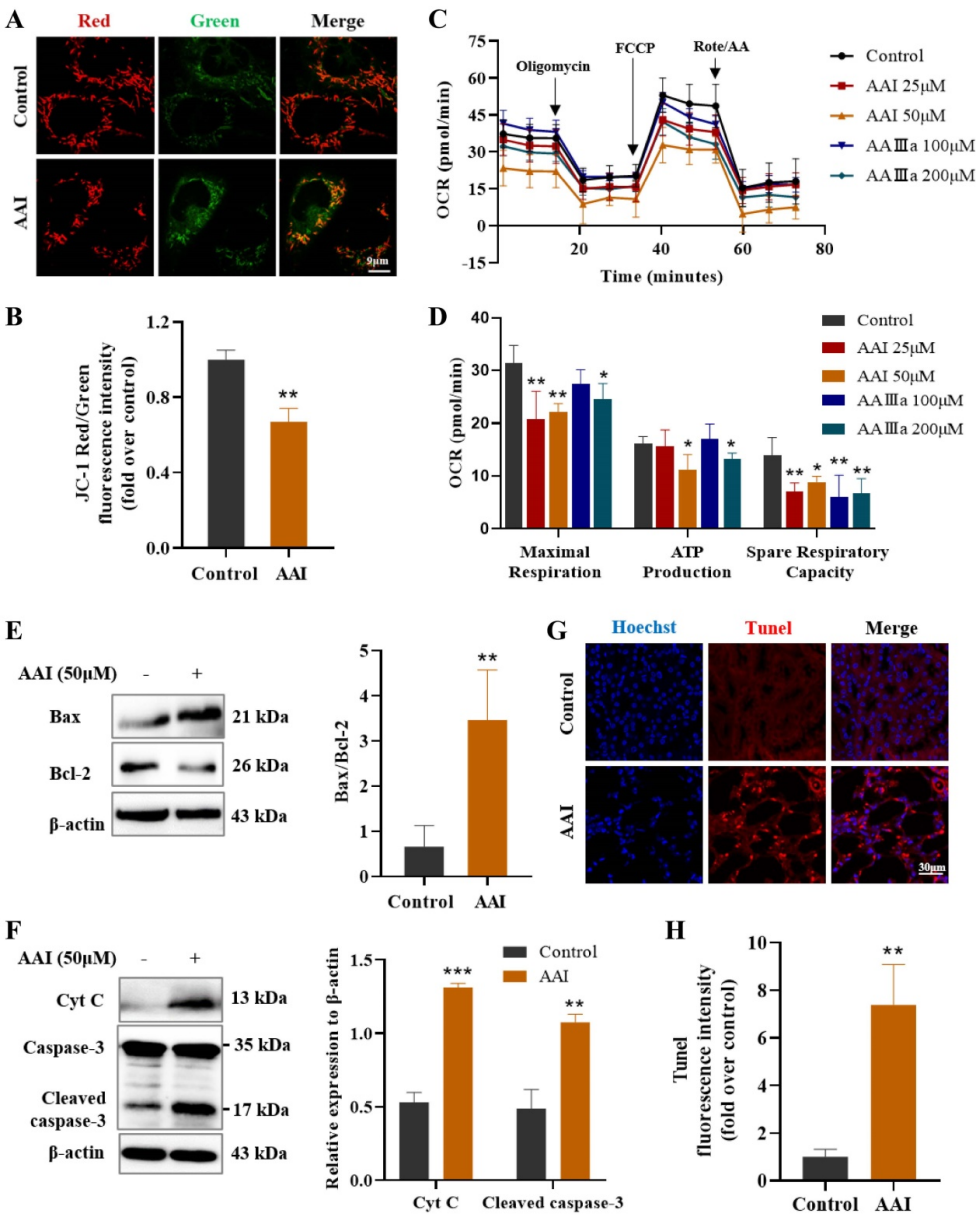
** Mitochondrial dysfunction caused by AAI-induced metabolism disorder and apoptosis.** (**A**) The JC-1 staining for mitochondrial membrane potential, scale bar = 9 µm. (**B**) Fluorescence intensity quantitative statistics correspond to Figure 5A. (**C**) The determination of oxygen consumption rate (OCR). (**D**) The indicators of OCR including maximal respiration, ATP production and spare respiratory capacity. (**E**) The indicators of mitochondrial apoptosis Bax and Bcl-2 proteins by western blotting and quantitative statistics. (**F**) The indicators of mitochondrial apoptosis Cyt C and caspase-3 proteins by western blotting and quantitative statistics. (**G**) Tunel staining in the kidneys of mice after AAI treatment, scale bar = 30 µm. (**H**) Fluorescence intensity quantitative statistics correspond to Figure 5G. n = 3, *P < 0.05, **P < 0.01, ***P < 0.001 compared with control.

Mitochondria is a central organelle for cellular metabolism and homeostasis, and mitochondrial dysfunction is closely associated with metabolic diseases and drug-induced organ injury 25, 26. Mitochondrial injuries had been found in AAN patients and animal models of AAN 27, 28. MMP is considered to be one of the most crucial indicators for evaluating mitochondrial damage. Here, we utilized the JC-1 detection kit to monitor MMP after treatment with AAⅠ. The red/green fluorescence intensity ratio significantly decreased via JC-1 staining (Figure 5A-B), suggesting that AAⅠ induced abnormalization of MMP in HK-2 cells.

To further evaluate the effects of AAⅠ and AAⅢa on the mitochondrial functions, we used an OCR experiment to detect mitochondrial function and cell metabolism in HK-2 cells. OCR is mainly caused by mitochondrial electron transfer and is commonly used to assess the mitochondrial oxidative phosphorylation function. Our results showed that both AAⅠ and AAⅢa effectively reduced basal and maximal respiration of HK-2 cells in a dose-dependent manner, while ATP production, spare respiratory capacity and non-mitochondrial oxygen consumption of HK-2 cells were significantly decreased after AAⅠ and AAⅢa treatment (Figure 5C-D, Figure S7A-C). Collectively, AAⅠ and its analogue AAⅢa induced mitochondrial dysfunction, thereby inhibiting cellular respiration and metabolism.

Mitochondrial dysfunction affects cell energy metabolism, and even induces cell apoptosis. We thus measured several indicators related with mitochondrial apoptosis by western blotting assay. AAⅠ remarkably up-regulated the expression of Bax and down-regulated the expression of Bcl-2 (Figure 5E). AAⅠ increased the release of cytochrome C (Cyt C) in the cytoplasm, suggesting that AAⅠ could cause changes in mitochondrial membrane permeability and loss of transmembrane potential. Subsequently, AAⅠ activated caspase-3 and caused mitochondrial apoptosis (Figure 5F). In addition, AAⅠ induced apoptosis of renal cells by TUNEL staining in AA-treated mice (Figure 5G-H). These results indicated that mitochondrial dysfunction induced by AAⅠ may result in metabolism disorder and mitochondrial apoptosis.

## Discussion

**Figure 6:**
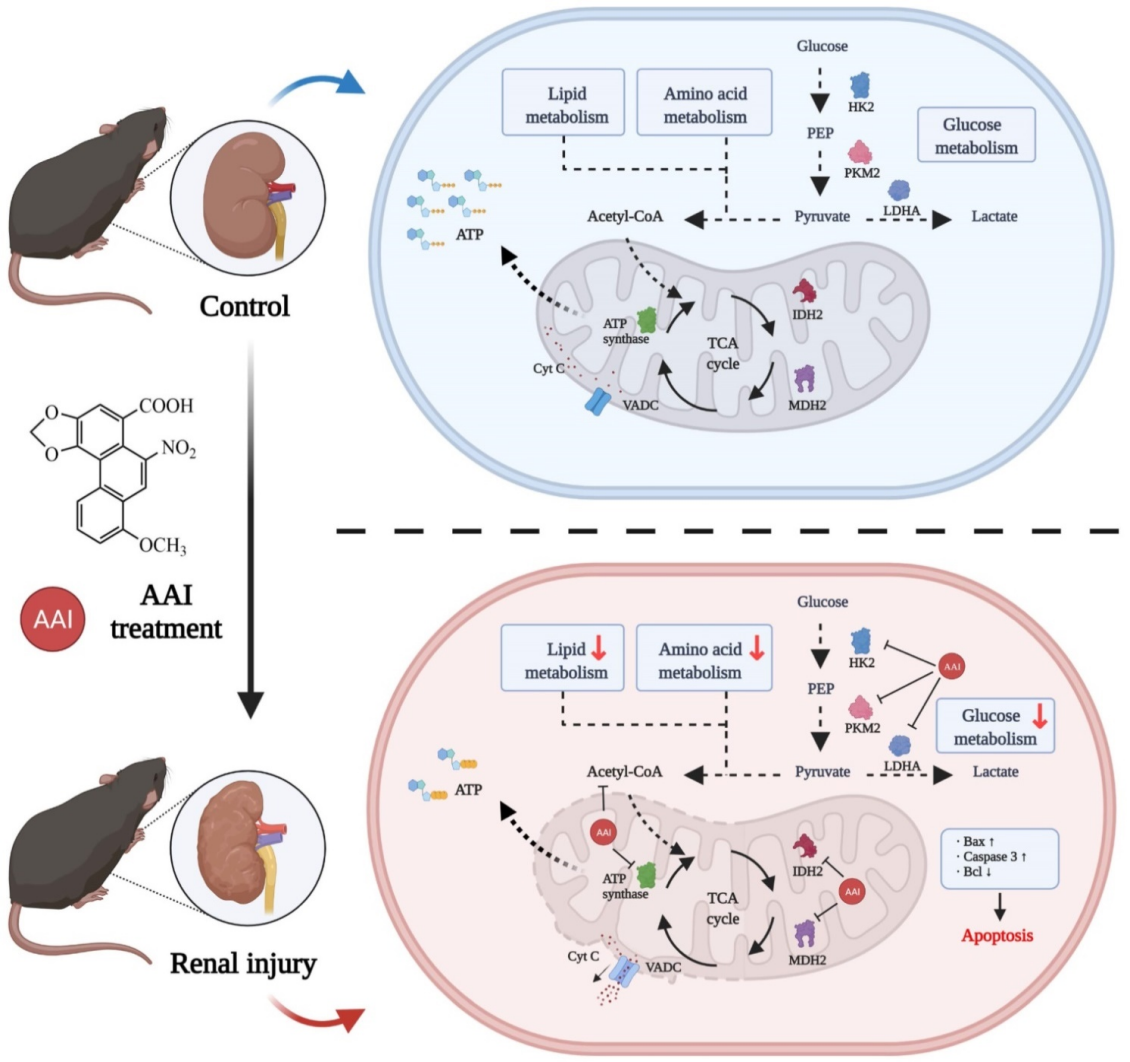
** Schematic model showing the molecular mechanisms of the AA-induced nephrotoxicity.** Activity-based protein profiling (ABPP) in combination with cellular thermal shift assay (CETSA), as well metabolomic studies showed that AAs directly target multiple key enzymes in the metabolic process such as lipid metabolism, amino acid metabolism, and TCA cycle, and then impairs mitochondrial dysfunction and induces apoptosis. Overall, our study provides novel insight into underlying mechanisms of AAs-induced kidney toxicity and pathogenesis.

AAs-induced AAN, characterized by interstitial nephritis, tubular atrophy and renal fibrosis, is a major global health problem 29. However, the direct binding protein targets of AAs in the kidney are missing and AA-induced nephrotoxicity is not fully understood. In this study, we utilized an ABPP combined with CETSA and metabolomic approaches to investigate AAs-binding proteins and characterized AAs-induced nephrotoxicity. Our results indicated that AAs can covalently target many proteins associated with metabolic processes and mitochondrial function, suggesting that the metabolic disorder and mitochondrial dysfunction induced by AAs results in nephrotoxicity (Figure 6).

Previously, AA-DNA adduct formation has been shown to mediate A>T transversions to induce genotoxicity and carcinogenesis 30, 31. The underlying mechanism is due to aristolochic acid bioactivated to an electrophilic cyclic aristolactam-nitrenium ion with delocalized positive charge, covalently binding to the exocyclic purine nucleotides of DNA 18, 32, 33. Here, we designed aristolochic acid probes, and then identified AA-targeting proteins via the ABPP method. Interestingly, our experimental results showed that the targeting mechanisms of actions of AAs are like an exploding bomb, which impaired multiple crucial metabolic processes and mitochondrial dysfunction and then caused renal cell apoptosis or death.

Recent studies have shown that metabolomics can identify and profile functional metabolites to reveal the varied pathological mechanisms of AAs-induced nephrotoxicity, which including the inhibition of amino acid metabolism, fatty acid metabolism, glucose metabolism and the TCA (Krebs) cycle 34-37. However, the targeting mechanism of action is unclear. Notably, our results indicated that AAs can directly target to certain key enzymes in the metabolic process including IDH2 and MDH2 (Krebs cycle) 38, PKM and LDH (aerobic respiration) 39, FASN (fatty acid beta-oxidation) 40, HK2 (glucose metabolism) 41 and ATP synthase (cellular respiratory chain). Subsequently, AAs targeting several vital enzymes were demonstrated by pull-down and cellular colocalizing image experiments. In addition, CETSA assays further validated that AAs could directly bind to IDH2, MDH2, PKM, PC, LDHA and HK2 proteins. Enzyme activity assays showed that AAs inhibited their enzymatic function and expression. Here, we further validated that AAs exposure results in aberrant metabolic pathways similar to the above approach via a metabolomic method. Although our study is useful for identifying AAs covalent targets, this experimental approach is inadequate for the identification of non-covalent or reversible binding proteins of aristolochic acid. It will be important to determine the function of targeting proteins in AA-induced nephrotoxicity in future studies.

In addition, AAs preferentially target PTECs in the kidney because of the selective reabsorption of AAs via organic anion transporters (OATs) 42. The mitochondrial enrichment and oxygen consumption of the kidney are second only to the heart 43. Our data also indicated that AAs target to multiple mitochondrial proteins and impair its biological functions. Indeed, AAs could lower MMP and reduce mitochondrial respiration chain and ATP production via JC-1 staining and seahorse experiments. It has been previously reported that AAs induce mitochondrial polarization and inhibit ATP production, thereby leading to apoptosis 28. Taken together, AAs can induce mitochondrial apoptosis via targeting mitochondrial proteins and impairing their homeostasis.

## Conclusions

In summary, for the first time, activity-based protein profiling (ABPP) in combination with bio-orthogonal click chemistry reaction and cellular thermal shift assay (CETSA), as well metabolomic studies revealed that AAI directly targeting multiple key enzymes in the metabolic process such as lipid metabolism, amino acid metabolism, aerobic respiration, and TCA cycle to impair mitochondrial dysfunction and induce apoptosis in the kidney. Overall, our studies provide novel insight into underlying AAs-induced kidney toxicity and pathogenesis of AAN, which is critical to developing therapeutic strategies for AAN.

## Materials and Methods

### Synthesis of aristolochic acid probe

All the reagents and solvents were purchased from Sigma-Aldrich and AK Scientific, and used without further purification unless stated otherwise. Reactions were monitored by thin-layer chromatography (TLC). Column chromatography was performed on silica gel 200~300 mesh. All ^1^H NMR (500 MHz), ^13^C NMR (125 MHz) spectra were recorded. ^1^H NMR Spectroscopy splitting patterns were designated as singlet (s), doublet (d), triplet (t), quartet (q). Splitting patterns that could not be interpreted or easily visualized were designated as multiplet (m).

A mixture of aristolochic acids (0.20 mmol), K_2_CO_3_ (41.4 mg, 0.30 mmol), propargyl bromide, 80% in toluene (0.20 mmol) in DMF (2 mL) was stirred at room temperature. Subsequently, the reaction was diluted with H_2_O (3 mL) and extracted with ethyl acetate (20 mL × 2). The organic phases were combined, washed with brine (5 mL × 3), dried anhydrous Na_2_SO_4_, and concentrated under vacuum. The residue was purified by flash column chromatography, which gave the aristolochic acids probes (**AP1** and **AP2**). ^1^H NMR, ^13^C NMR and HRMS of aristolochic acids probes were showed in Supporting information.

### Cell culture and viability

Human renal tubular epithelial cell line (HK-2) was obtained from American type culture collection and grown in DMEM/F12 medium (supplemented 10% fetal bovine serum and 1% penicillin/streptomycin), and cultured at 37 ℃ and 5% CO_2_ in a humidified incubator. HK-2 cells were cultured in a 96-well plate for 24 h and treated with different concentrations of aristolochic acid I (AAⅠ) sodium salt, its analogues (AAⅣa and AAⅢa) and AA-probes (AP1 and AP2) for 48 h. The CCK-8 kit was used to detect cell viability.

### Animal experiments

Animal experiments were approved by the Care and Use of Laboratory Animals Center of Shenzhen People's Hospital. C57 BL/6 wild-type mice (male, 20 ± 2g, 7-8 weeks old) were treated with aristolochic acid I (2 mg/kg, once a day for 4 weeks) by intraperitoneal injection (i.p.). In the control group, mice were administrated with normal saline buffer. After 4 weeks, all mice were anesthetized to collect blood and kidney.

### Blood biochemical analysis

Serum creatinine (CRE) and urea (UREA) were determined by an automatic biochemistry analyzer (TOSHIBA, Japan). Blood routine test was measured by an automatic blood analyzer according to the manufacturer's protocols.

### Renal histological analysis

Kidney tissues were embedded in paraffin and cut into 4 μm sections for hematoxylin-eosin (H&E) staining to evaluate changes in histological morphology.

### Enzyme activity assay

The biological activities of ATP synthase (Abcam, UK) and pyruvate kinase M 1/2 (PKM) (Abcam, UK) were monitored according to the manufacturer's protocols of kits.

### Mitochondrial function and cellular oxygen consumption

The mitochondrial membrane potential (MMP) was determined by using MitoScreen (JC-1 staining) kit (BD biosciences, USA). According to the red/green fluorescence intensity ratio, MMP was evaluated in HK-2 cells. In addition, cellular respiration and mitochondrial function were detected by an oxygen consumption rate (OCR) experiment by using a Seahorse Extracellular Flux Analyzer (Seahorse Bioscience, USA).

### Western blot assay

Crude proteins were extracted from HK-2 cells treated with AAⅠ. Western blot assay was carried out as previously reported 20. The primary antibodies were used anti-Caspase 3 (Proteintech, USA), anti-Bax (Proteintech, USA), anti-Bcl-2 (Proteintech, USA), anti-Cyt C (Proteintech, USA) and anti-β-actin (Affinity biosciences, China). The protein bands were quantified by ImageJ software, and normalized to the corresponding to the β-actin level.

### Fluorescence imaging

To track the cellular location of the AA-probe, fluorescence imaging studies were performed. HK-2 cells were seeded in the 4-chamber dishes and incubated with AA-probe at different concentrations with or without original compounds. After incubation for 4 h, fluorescence imaging was implemented as previously described 44. For co-localization of AA-probe and target proteins, immunofluorescence staining was carried out based on probe imaging *in situ*. The primary antibodies were anti-IDH2 (Abcam, UK), anti-ATP synthase (Abcam, UK), anti-MDH2 (Abcam, UK) and anti-PKM (Proteintech, USA). Finally, cellular imaging was acquired with a confocal fluorescence microscopy (Leica TCS-SP8-SR, Germany).

### Fluorescence labeling experiments

For labeling protein *in situ* and* ex vivo*, a fluorescence labeling experiment was performed as previously reported 21, 45. HK-2 cells *in situ* were treated with AA-probes in the absence or presence of competitors, proteins were extracted. For the lysate of the kidney *ex vivo*, proteins first extracted were incubated with probes or competitors. Samples were clicked with a fluorescence dye. Equal amounts of samples were separated by SDS-PAGE gel electrophoresis and analyzed by a fluorescence imaging scanner (Azure Sapphire RGBNIR, USA).

For labeling protein *in vivo*, the kidneys of mice were collected after the tail vein injection with AP2. Then proteins extracted from the kidney were clicked with a fluorescent dye. Next, experimental procedures were as above described.

### Target identification based on LC-MS/MS

Pull-down and LC-MS/MS experiments were used to identify target proteins of AA. As above described, HK-2 cells and kidney lysates first were incubated with probe or DMSO for 4 h. Protein samples were conjugated with biotin-azide by a clicked reaction. Subsequently, samples were incubated with beads, eluted, reduced, alkylated, and trypsinized to form peptides that were desalted 20. Ultimately, peptides were labelled by using TMT 10 plex Mass Tag reagents (Thermo, USA) and analyzed by LC-MS/MS (Orbitrap Fusion Lumos, Thermo, USA).

For pull down-western blot assay, the target proteins were validated by western blot assay, procedures were as previously described 20.

### Target protein data analysis

According to the TMT protein intensity of DMSO (control) group and AP2 (probe) group. 912 identified target proteins in mice kidney lysis solution were converted into being 860 targets of human homologous genes for further analysis 46. The differential target proteins were selected basis of the absolute fold change > 1.2 and P value < 0.05. Selected proteins were subjected to the heatmap and Volcano plots analysis. Moreover, Veen and GO enrichment analyses were performed on the selected proteins.

### Metabolomic analysis by LC-MS/MS

For the extraction of metabolites in kidney tissue and serum, sample preparation was as previously described 47, 48. Subsequently, both kidney and serum samples were filtered and then analyzed by LC-MS/MS.

For data analysis, the original file was processed using the Compound Discoverer 3.1 software to perform peak alignment, peak picking, and quantitation for each metabolite by mzCloud, mzVault and MassList database. Metabolite statistical analyses were performed using the MetaboAnalyst 5.0 web servers and statistical software R (R version R-4.1.1). In chemometrics analysis, Principal components analysis (PCA) and Orthogonal Partial Least Squares-Discriminant Analysis (orthoPLS-DA) were used to discriminate the sample distribution tendency between AAI treatment and control groups. Volcano plots were used to highlight the significant differential metabolites based on log2(FC) and -log10(FDR) and VIP value of metabolites by ggplot2 in R language. The significant differential metabolites which have HMDB classification annotation were used to over representation analysis. The metabolic pathways enrichment of differential metabolites was performed using the KEGG database.

### Cellular Thermal Shift Assay (CETSA)

Cellular thermal shift assay (CETSA)-western blot (WB) assay 49 was carried out to further validate AAs' binding to target proteins. Proteins were extracted from HK-2 cells, incubated with AA or DMSO, and then heated by CETSA heat pulse procedure (Applied biosystems, Thermo, USA). Samples were detected by western blot.

### Molecular docking analysis

The 3D structure file of AAⅠ was downloaded from PubChem. The structures of IDH2 (PDB: 1AI2), MDH2 (PDB: 4WLF), PKM (PDB: 4GLN), LDHA (PDB: 5W8J), HK2 (PDB: 2NZT), VDAC1 (PDB: 6G6U), ATP synthase (PDB: 5TSJ) and FASN (PDB: 2PX6) were obtained from RCSB PDB. The AutoDock Tool was used for preparing structures of the ligand and protein. Subsequently, the AutoDock Vina was employed for docking AAⅠ to these proteins. Pyrx-0.8 was utilized for molecular docking. All docking results were analyzed and visualized by Pymol.

### Statistical analysis

All data were described as mean ± SEM values for at least 3 independent experiments. Experimental statistical analysis was performed by one-way ANOVA followed by the Tukey's test in multiple groups. An unpaired two-tailed t-test was carried out in two groups. All statistical analysis was calculated in GraphPad Prism 8.0 software.

## Supplementary Material

Supplementary figures.

## Figures and Tables

**Figure A:**
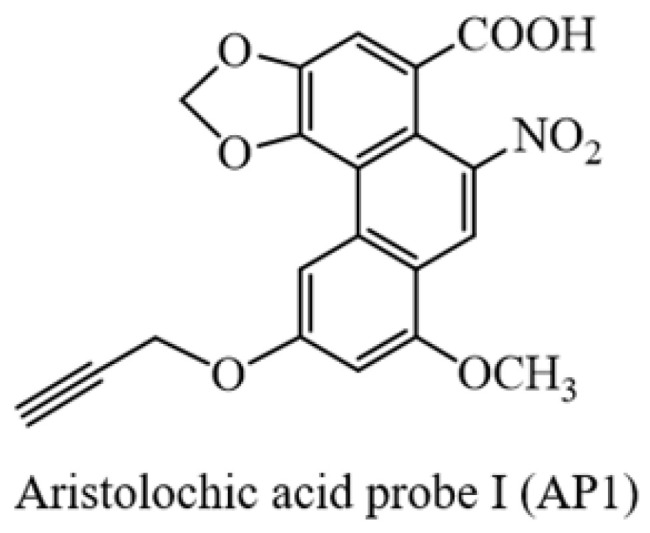
Yellow solid (yield: 59%); ^1^H NMR (500 MHz, DMSO-*d_6_*) δ 10.82 (s, 1H), 8.57 (s, 1H), 8.11 (s, 1H), 7.77 (s, 1H), 6.86 (s, 1H), 6.51 (s, 2H), 4.83 (s, 2H), 4.02 (s, 3H), 3.62 (s, 1H); ^13^C NMR (125 MHz, DMSO-*d_6_*) δ166.1, 162.3, 158.8, 147.5, 146.1, 142.3, 132.2, 121.8, 118.4, 117.1, 112.9, 112.6, 104.3, 103.6, 100.4, 78.8, 78.3, 56.7, 53.2; HRMS m/z: [M-H]^-^ Calcd for C_20_H_12_NO_8,_ 394.0568, Found 394.0563.

**Figure B:**
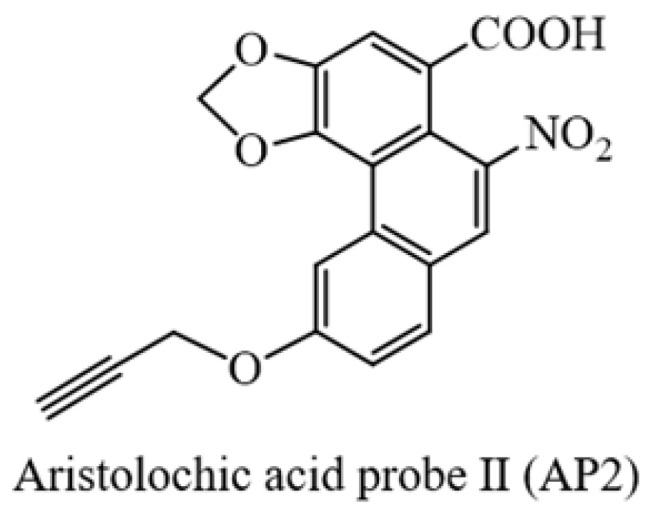
Yellow solid (yield: 54%); ^1^H NMR (500 MHz, DMSO-*d_6_*) δ 10.70 (s, 1H), 8.59 (s, 1H), 8.49 (s, 1H), 8.13 (d, *J* = 12 Hz, 1H), 7.77 (s, 1H), 7.31 (d, *J* = 12 Hz, 1H), 6.53 (s, 2H), 4.84 (s, 2H), 3.63 (s, 1H); ^13^C NMR (125 MHz, DMSO-*d_6_*) δ166.1, 160.6, 147.4, 146.1, 143.1, 133.2, 131.5, 127.9, 122.0, 121.8, 119.5, 117.9, 117.1, 112.6, 111.6, 103.6, 78.8, 78.3, 53.2; HRMS m/z: [M-H]^-^ Calcd for C_19_H_10_NO_7,_ 364.0463, Found 364.0450.

## Abbreviations

AA: aristolochic acid
AAN: aristolochic acid nephropathy
ABPP: activity-based protein profiling
CETSA: cellular thermal shift assay
OCR: oxygen consumption rate
CHN: Chinese herb nephropathy
BEN: Balkan endemic nephropathy
UTUC: upper tract urothelial cancer
TCM: traditional Chinese medicine
PTEC: proximal tubular epithelial cells
MMP: mitochondrial membrane potential
GO: gene ontology
TCA: tricarboxylic acid
PPI: protein and protein interaction
IDH2: isocitrate dehydrogenase 2
MDH2: malate dehydrogenase 2
PKM: pyruvate kinase M1/2
LDH: lactate dehydrogenase A/B
PC: pyruvate carboxylase
VDAC1: voltage-dependent anion-selective channel protein 1
ACACA: acetyl-CoA carboxylase 1
FASN: fatty acid synthase
HK2: hexokinase-2
CRE: creatinine
QC: quality control
PCA: principal component analysis
Ortho PLS-DA: Orthogonal partial least squares-discriminant analysis
HMDB: human metabolome database
Cyt C: cytochrome C
OATs: organic anion transporters
