# Supplementary material for: Dissection of Targeting Molecular Mechanisms of Aristolochic Acid-induced Nephrotoxicity via a Combined Deconvolution Strategy of Chemoproteomics and Metabolomics
Source: Int J Biol Sci. 2022 Feb 21;18(5):2003–17. doi: 10.7150/ijbs.69618 (PMC8935225; doi:10.7150/ijbs.69618)
Supplement: Supplementary file 1 — Supplementary figures. [file ijbsv18p2003s1.pdf]

Supplementary materials

Dissection of Targeting Molecular Mechanisms of Aristolochic Acid-induced Nephrotoxicity via a Combined Deconvolution Strategy of Chemoproteomics and Metabolomics

Qian Zhang[a, b, c, #], Piao Luo[a, c, #], Jiayun Chen[a, #], Chuanbin Yang[d, #], Fei Xia[a], Junzhe Zhang[a], Huan Tang[a], Dandan Liu[a], Liwei Gu[a], Qiaoli Shi[a], Xueling He[a], Tong Yang[a], Jigang Wang[a, b, c, d, e, f, g, *]

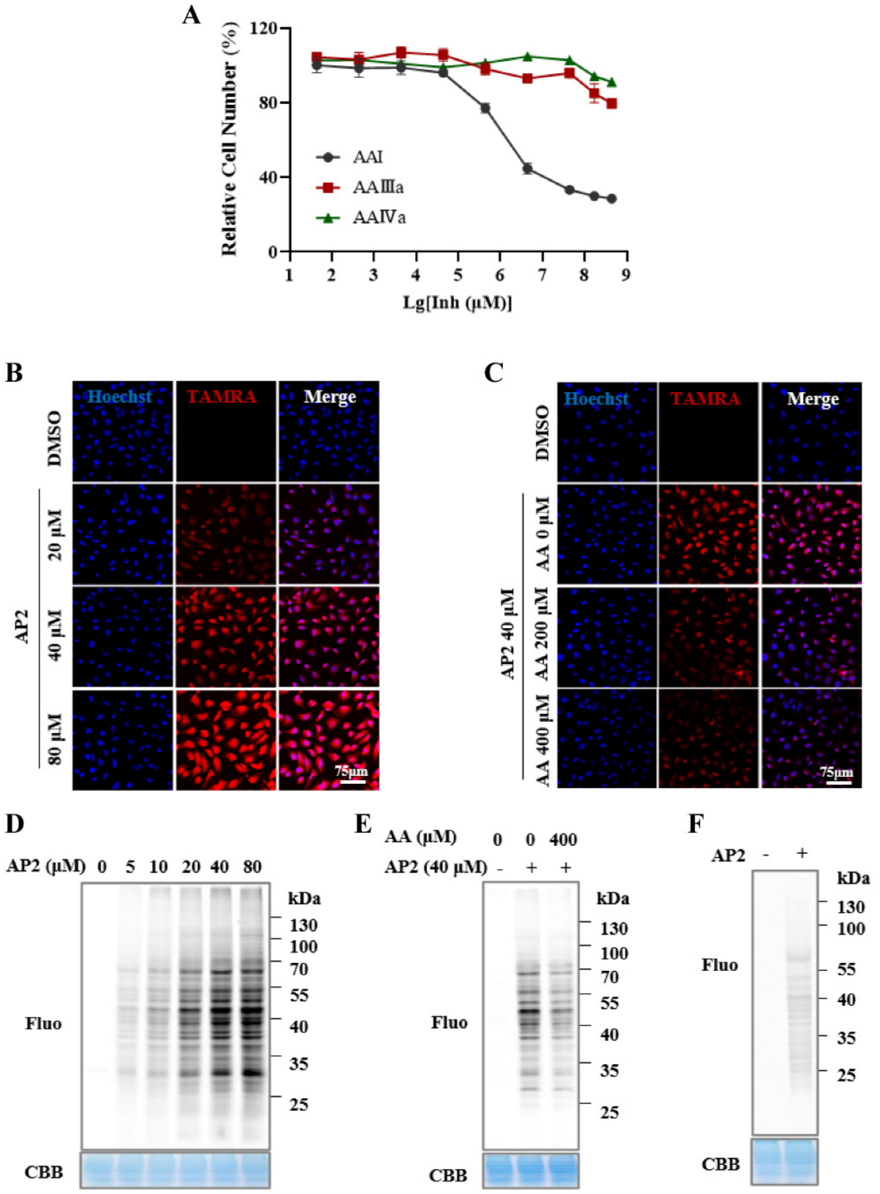

Figure S1. Fluorescence labeling and cellular imaging of AA-probe. (**A**) The inhibition effects of aristolochic acid I (AAI) and two analogues (AAIVa, AAIIIa) on HK-2 cell. (**B-C**) fluorescence labelling protein of cellular imaging location of the AP2 (40 μM) in HK-2 cells (scale bar = 75 μm). (**D**) *Ex vivo* labelling protein in an AP2 dose-dependent manner in the lysate of the kidney. (**E**) The competition of labelling protein with AP2 by excess AAI in the lysate of the kidney. (**F**) *In vivo* fluorescence labelling protein of AP2.

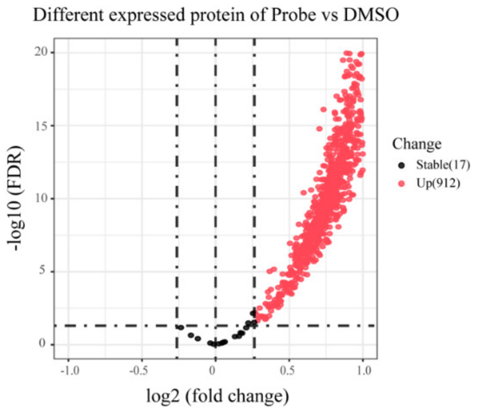

Figure S2. Aristolochic acid target proteins in the lysate of the kidney. Volcano plot depicting the differential enrichment of proteins in AP2 vs DMSO groups.

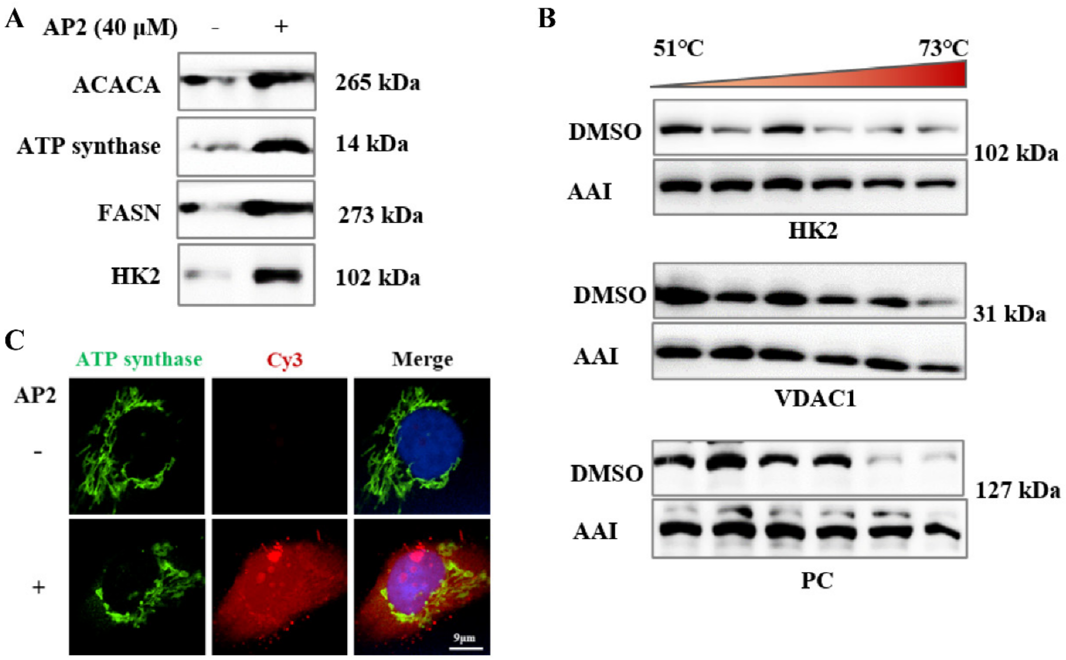

Figure S3. Validation of aristolochic acid other targets (**A**) Pull-down Western blotting study to verify AA directly targeting to ACACA, ATP synthase, FASN and HK2 proteins. (**B**) CETSA-Western blotting experiment to validate the AA binding to HK2, VDAC1 and PC proteins. (**C**) Immunofluorescence staining of target proteins (green) and AA-probe (a red fluorescence dye), scale bar = 9 μm.

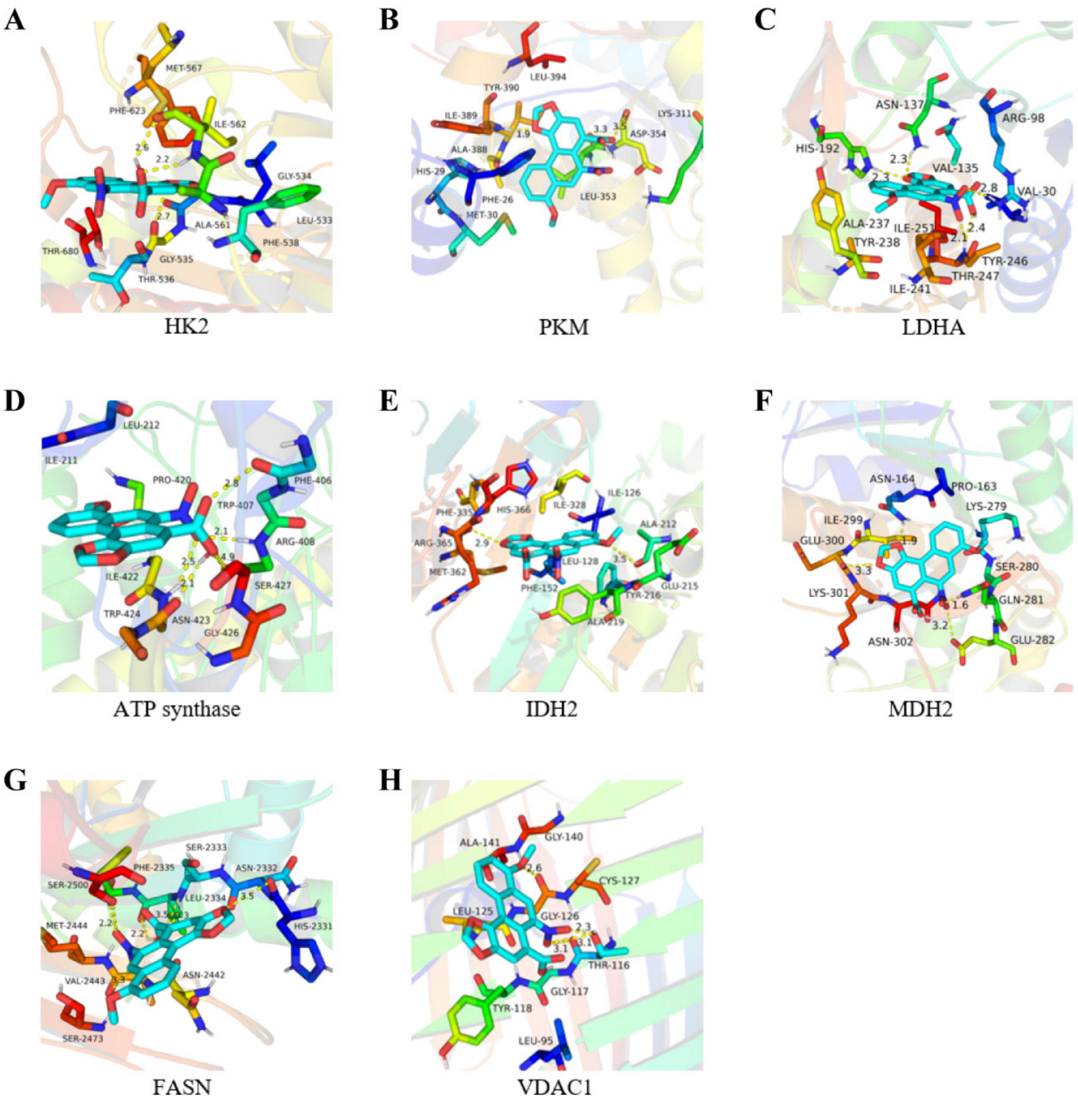

Figure S4. Binding model of AAI with HK2 (**A**), PKM (**B**), LDHA (**C**), ATP synthase (**D**), IDH2 (**E**), MDH2 (F), FASN (**G**) and VDAC1 (**H**) by docking analysis. Yellow dotted lines represent hydrogen bonds.

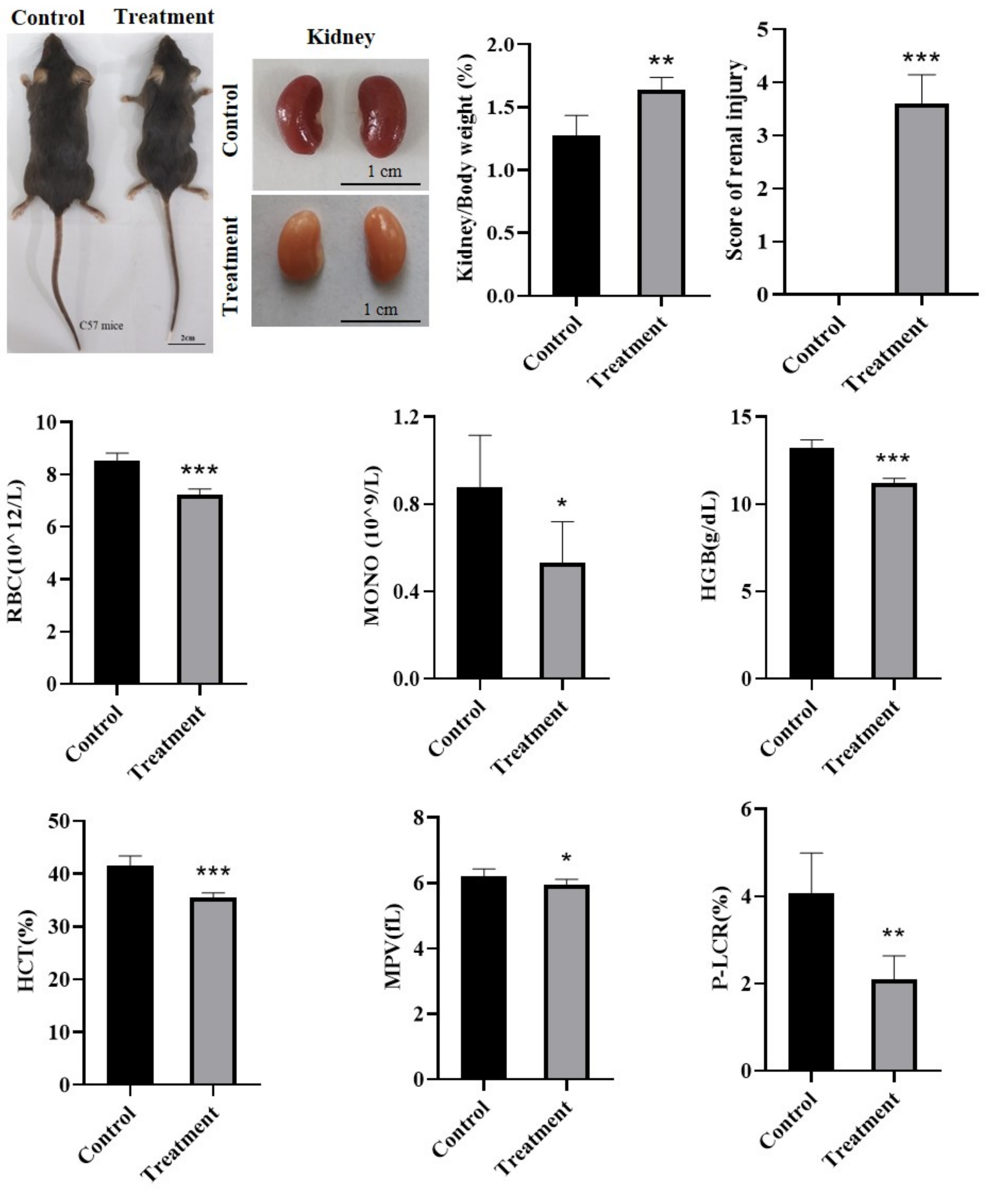

Figure S5. Relative parameters of AAI-treated mice. (A) Representation of the appearance of mice and kidneys. (**B**) The kidney/body weight ratio and the score of the pathology changes of kidney from AA-treated and control mice. (C) The parameters of routine blood tests. All data represent means ± SEM, n≥3, *p<0.05, **p<0.01, ***p<0.001 model (AA treatment) vs control.

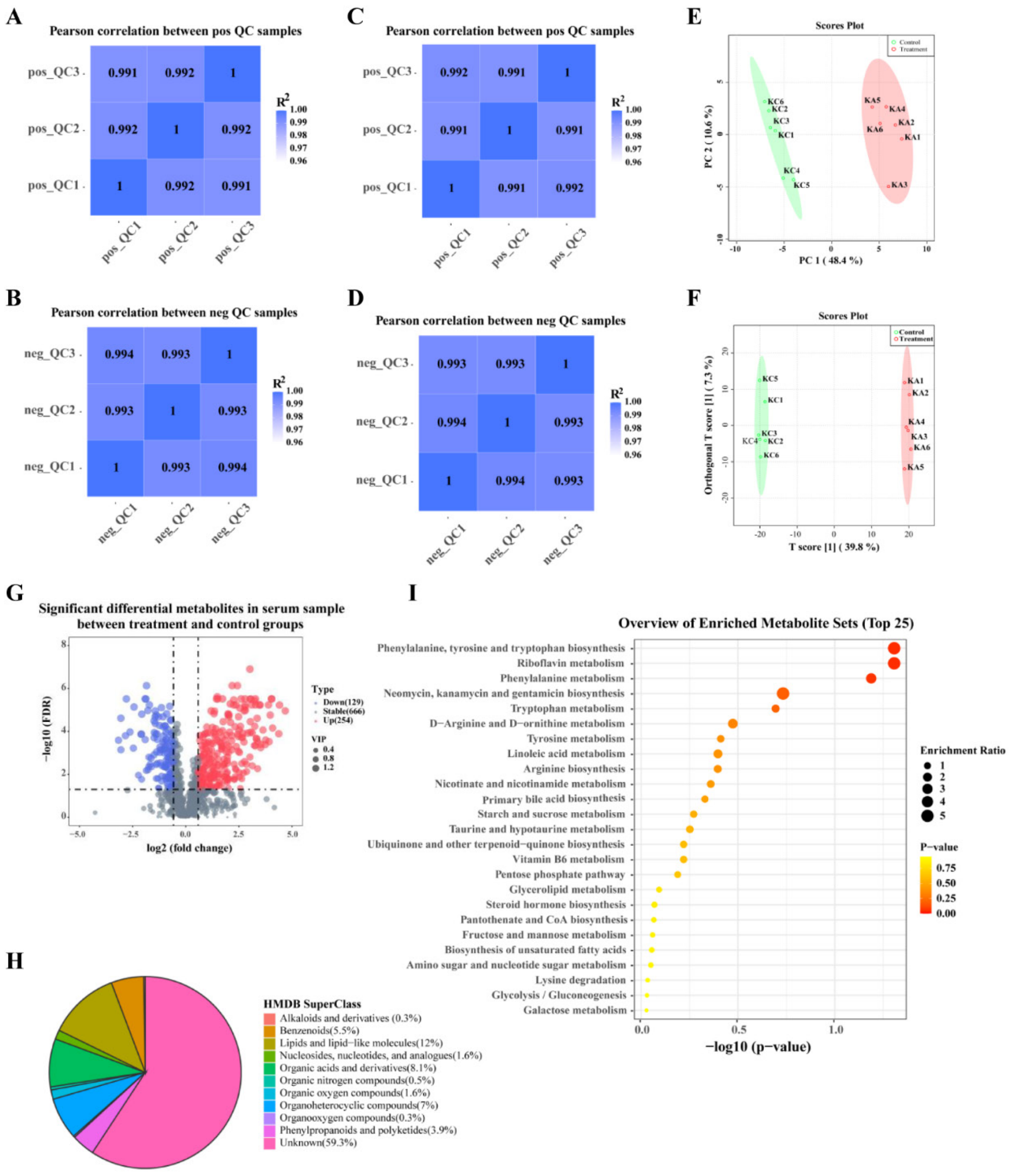

Figure S6. Metabolomic analysis of AA-induced nephrotoxicity. (**A-B**) The correlation analysis of samples QC in kidney sample. (**C-D**) The correlation analysis of samples QC in the serum sample. (**E**) The PCA plots of the DMSO and treatment groups in the serum sample. (**F**) Ortho PLS-DA analysis of the DMSO and treatment groups in the serum sample. (**G**) The volcano map displays the different metabolites in serum. Up-regulated metabolites were represented by red dots, down-regulated metabolites were represented by blue dots. (**H**) Classification information of HMDB database annotations in serum. (**I**) KEGG biochemical metabolic pathway and signal transduction pathway in serum.

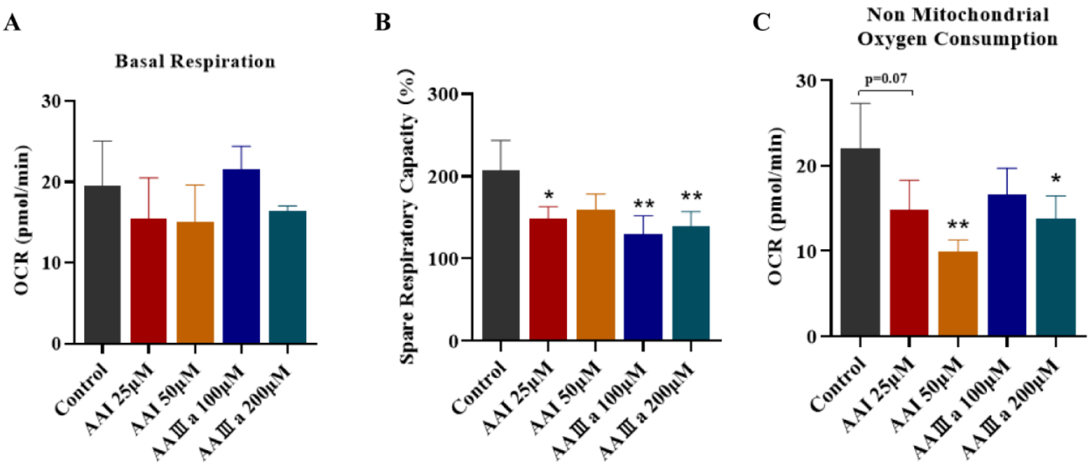

Figure S7. The effect of AAI on cellular respiration and metabolism. (**A-C**) The indicators of oxygen consumption rate (OCR) include basal respiration, spare respiratory capacity (%) and non-mitochondrial oxygen consumption. n = 3, $*P < 0.05$, $**P < 0.01$ compared with control.

^{1}H NMR, ^{13}C NMR and HRMS of aristolochic acids probes (AP1 and AP2)

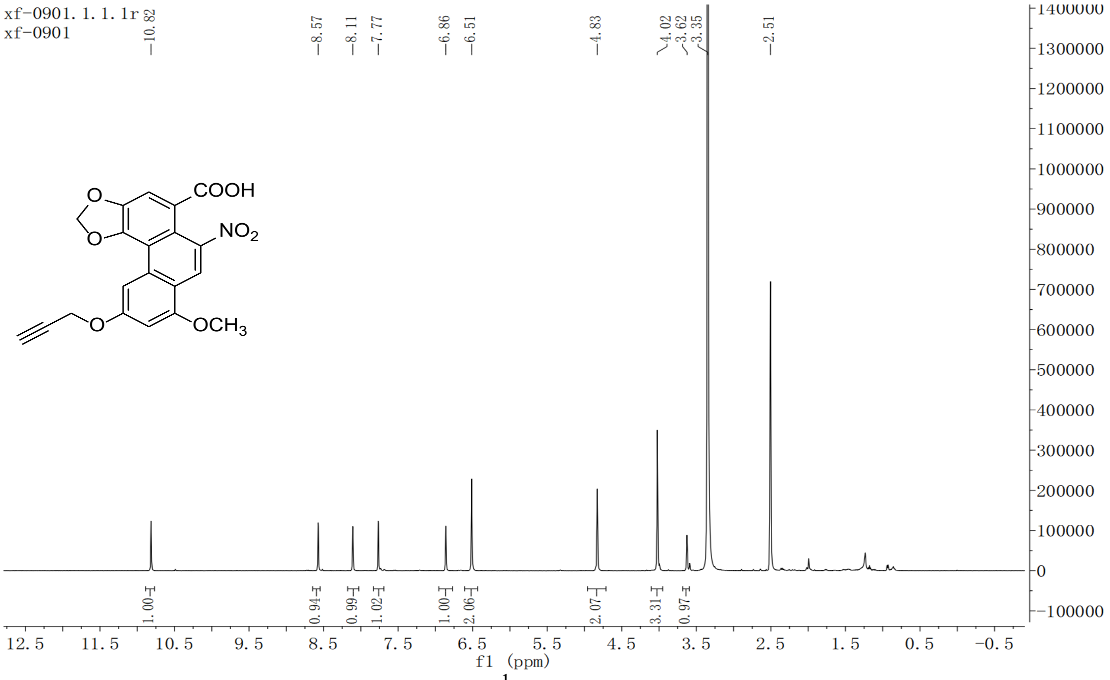

^{1}H NMR of **AP1**

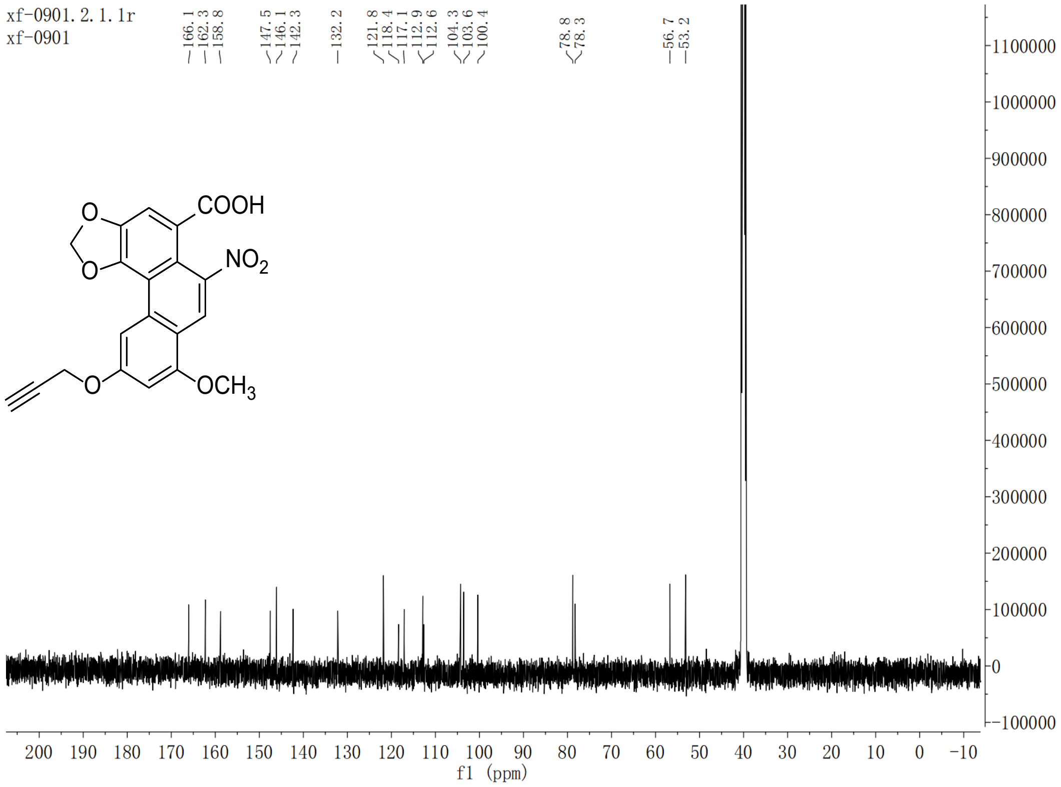

^{13}C NMR of **AP1**

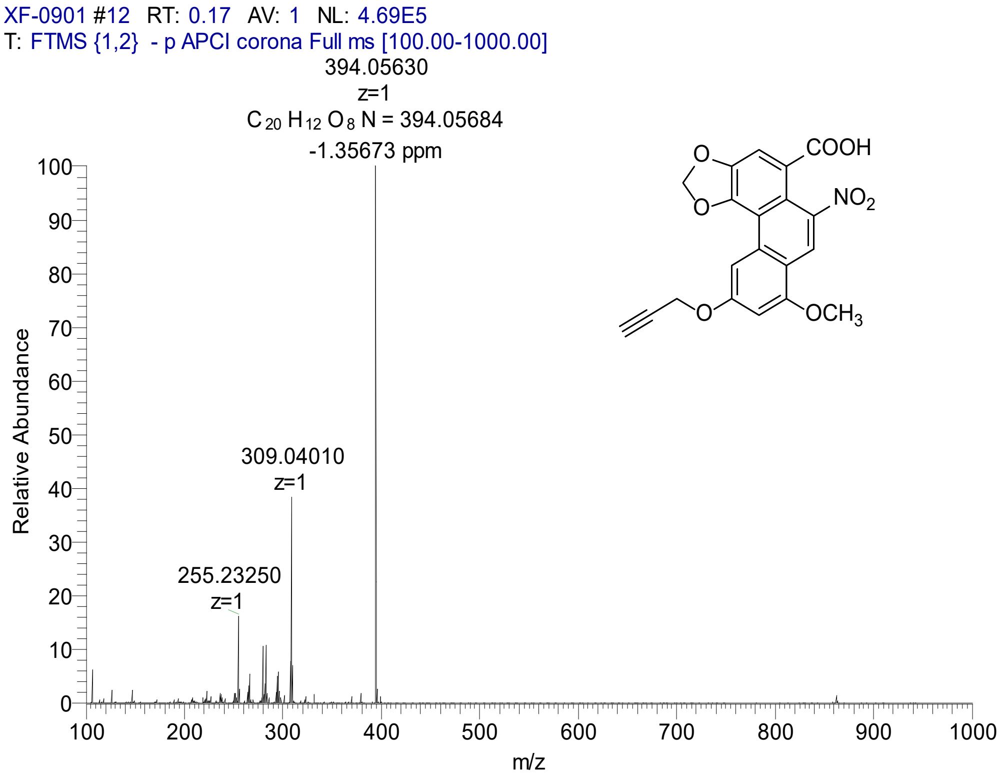

HRMS of **AP1**

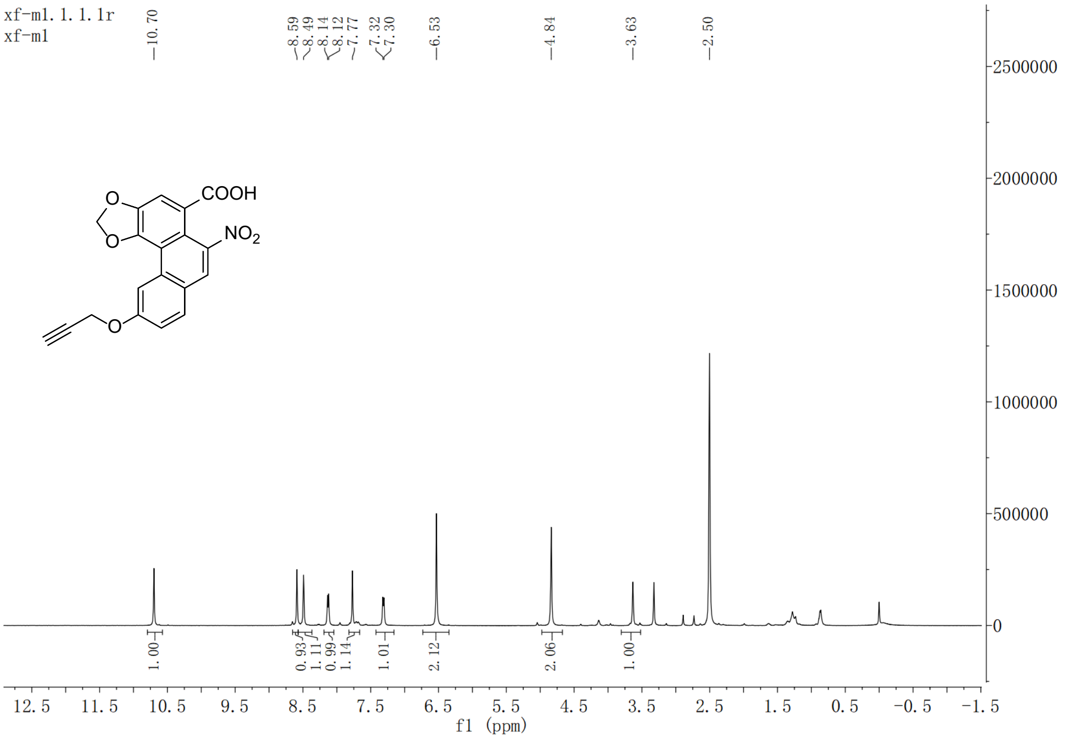

^{1}H NMR of **AP2**

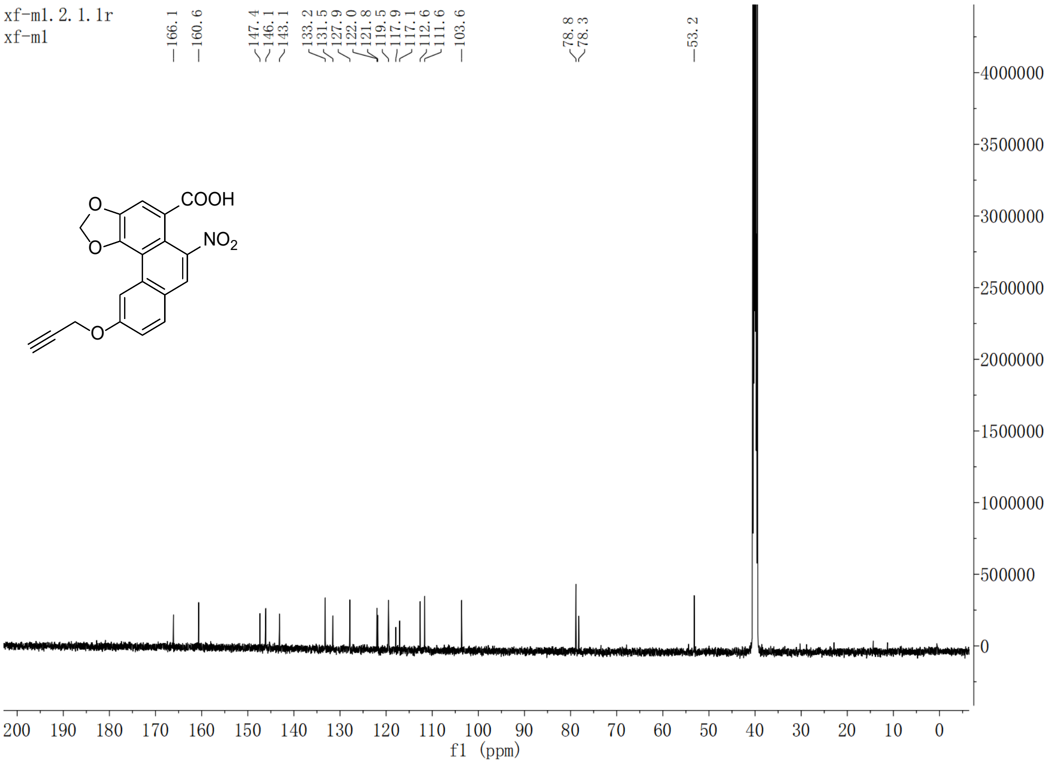

^{13}C NMR of **AP2**

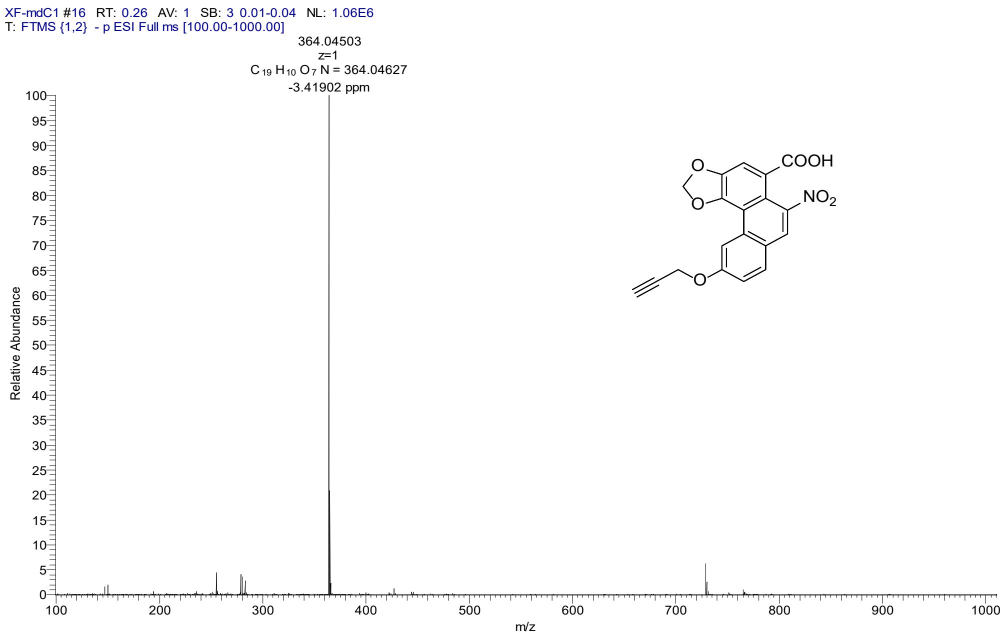
XF-mdC1 #16 RT: 0.26 AV: 1 SB: 3 0.01-0.04 NL: 1.06E6
T: FTMS {1,2} - p ESI Full ms [100.00-1000.00]
364.04503
z=1
C 19 H 10 O 7 N = 364.04627
-3.41902 ppm
Relative Abundance
m/z
COOH
NO2
O

HRMS of **AP2**
